# Feature Fusion of a Deep-Learning Algorithm into Wearable Sensor Devices for Human Activity Recognition

**DOI:** 10.3390/s21248294

**Published:** 2021-12-11

**Authors:** Chih-Ta Yen, Jia-Xian Liao, Yi-Kai Huang

**Affiliations:** 1 Department of Electrical Engineering, National Taiwan Ocean University, Keelung City 202301, Taiwan; 2 Department of Electrical Engineering, National Formosa University, Yunlin County 632, Taiwan; 10865120@gm.nfu.edu.tw (J.-X.L.); 10865122@gm.nfu.edu.tw (Y.-K.H.)

**Keywords:** wearable device, human activity recognition (HAR), inertial sensor, deep-learning, convolutional neural network (CNN), feature fusion

## Abstract

This paper presents a wearable device, fitted on the waist of a participant that recognizes six activities of daily living (walking, walking upstairs, walking downstairs, sitting, standing, and laying) through a deep-learning algorithm, human activity recognition (HAR). The wearable device comprises a single-board computer (SBC) and six-axis sensors. The deep-learning algorithm employs three parallel convolutional neural networks for local feature extraction and for subsequent concatenation to establish feature fusion models of varying kernel size. By using kernels of different sizes, relevant local features of varying lengths were identified, thereby increasing the accuracy of human activity recognition. Regarding experimental data, the database of University of California, Irvine (UCI) and self-recorded data were used separately. The self-recorded data were obtained by having 21 participants wear the device on their waist and perform six common activities in the laboratory. These data were used to verify the proposed deep-learning algorithm on the performance of the wearable device. The accuracy of these six activities in the UCI dataset and in the self-recorded data were 97.49% and 96.27%, respectively. The accuracies in tenfold cross-validation were 99.56% and 97.46%, respectively. The experimental results have successfully verified the proposed convolutional neural network (CNN) architecture, which can be used in rehabilitation assessment for people unable to exercise vigorously.

## 1. Introduction

With the popularization of wearable devices and reductions in their size and cost in recent years, sensors have been applied in human activity recognition (HAR). HAR is critical to promoting daily life development. HAR data are fed into algorithms, thereby allowing goals of monitoring, analysis, and assisting humans to be achieved [1]. For example, in fields such as sports training, medicine, and motion sensing [2,3,4,5,6,7,8,9,10,11], various sensors have been used to collect data on human activities, for example, from human computer interaction to surveillance, security, and health monitoring systems. Despite ongoing efforts in the field, the research addresses that activity recognition is still a difficult task in an unrestricted environment and faces many challenges [12]. Wearable devices, commonly worn on the waist, thigh, or wrists, collect activity data at regular intervals. Multiple sensors such as accelerometers, gyroscopes, and magnetometers are used to collect data on human postures, activities, or positions. The data are then preprocessed through denoising and normalization, among other procedures, for subsequent feature extraction and for training HAR classifiers [13]. The research gave the results; it is found that with a single sensor condition, the sensor has the highest accuracy when worn on the waist. Hence, we placed the sensor on the waist for data collection, and the data were preprocessed by data normalization. Then, it was used for subsequent feature extraction and training classifiers for related activity recognition.

In recent years, numerous researchers have used public datasets or databases for research and verification. One notable example is the Human Activity Recognition Using Smartphones Dataset provided by the University of California, Irvine (UCI). The dataset is hereafter referred to as the UCI dataset. With the rapid development of deep learning, an increasing number of researchers has employed it in feature extraction and activity recognition. Various studies have used sensors to collect data and analyze results for developing effective HAR systems using deep-learning techniques [14]. Yen et al. proposed a motion recognition algorithm based on a one-dimensional convolutional neural network (CNN), which they applied to self-recorded data and the UCI dataset for model verification and calibration. Application to the UCI dataset yielded an accuracy of 95.99% [15]. Xia et al. proposed a deep neural network that integrated long short-term memory (LSTM) and a CNN. That model replaced fully connected layers with global average polling to reduce the number of model parameters. Batch normalization was conducted to accelerate convergence. Application of the model to the UCI dataset resulted in an accuracy of 95.78% [16]. Mekruksavanich and Jitpattanakul proposed a four-layer CNN for feature extraction. An LSTM-based model was subsequently run. Data were generated using overlapping and nonoverlapping temporal windows, which had accuracies of 99.39% and 98.76%, respectively (as revealed through tenfold cross-validation) [17]. Yang et al. used an improved DenseNet model, in which the exponential linear unit function, batch normalization, and dropout technique were employed to prevent overfitting and the vanishing gradient problem, as well as to accelerate conversion. The UCI dataset, used for training and verification, had an accuracy of 95.89% [18]. Mutegeki and Han proposed a hybrid structure comprising one CNN layer and one LSTM layer. The UCI dataset was used for training and verification and yielded an accuracy of 92.13% [19]. Nidhi et al. proposed a multi-input network structure, in which kernels of varying size were employed in feature extraction. A gated recurrent unit (GRU) was then used to obtain the time sequence correlation. Applied to the UCI dataset, its accuracy was 96.20% [20]. Hernández et al. applied a bidirectional LSTM to the UCI dataset for training and verification. Models with three-layer network structures and no more than 175 units achieved favorable results; the accuracy was 92.67% [21]. Thakur and Biswas proposed the integration of automatic learning with CNNs and of manual feature attraction with expert knowledge. The statistical features of the time and frequency domains achieved a 99.1% accuracy in the UCI dataset [22]. Ullah et al. proposed a five-layer LSTM network structure, in which L2 regularization was used to prevent overfitting. Training and verification were conducted on the UCI data, yielding an accuracy of 93.13% [23]. Nafea et al. proposed using CNNs of varying kernel size to extract spatial information and using a bidirectional LSTM to obtain temporal information. The obtained spatiotemporal data were concatenated in a mixed model that was trained and verified using the UCI dataset, yielding a 97.05% accuracy [24]. Xu et al. developed InnoHAR, a deep neural network. By stacking inception modules, kernels of varying size were used for feature extraction. Subsequently, GRU was used to obtain time sequence correlation data. In the UCI dataset, an accuracy of 94.5% was achieved [25]. Avilés-Cruz presented coarse, moderate, and fine feature extraction networks. By using varying numbers of convolutional layers and max pooling layers, the output of the three models was made consistent. Next, the three models were concatenated, and the fully connected layer was used for subsequent motion classification. In the UCI dataset, the accuracy reached 100% [26].

The wearable technique uses sensing devices to be mounted on the subject to collect data from the sensors. As human activity contains actions of different bodily positions, the research of human activity needs to capture information from more than one sensor installed on the different parts of the body of the person. The sensors can be installed on more parts of the subject, such as head, hands, and feet, to collect more data and use data fusion technology with a convolutional neural network to recognize more movements. Wearable devices must be designed with user accessibility in mind. Lightweight, modern, and comfortable wearing devices with embedded sensors are used for activity monitoring. This study used the UCI dataset to verify the proposed model. The collected data by the authors were classified in the same model. A feature fusion model with kernels of varying size was developed. The use of different kernel sizes enabled the determination of local correlated features of different lengths in the data. This multidimensional feature extraction process increased the accuracy of HAR. The wearable device was positioned on participants’ waists. The collected data were analyzed using an HAR algorithm. The algorithm used a CNN to achieve feature recognition and to classify and identify six human daily activities. In conclusion, the main contribution of this paper is to propose multi-scale feature extraction through multi-scale parallel convolutional neural networks, thereby improving the accuracy of human activity recognitions. As the same CNN algorithm is used in different inertial sensors (i.e., Samsung Galaxy S2 for UCI database and MPU-6050 in our experiment), it can still obtain high recognition performance. The wearable device that collects data at the waist can be more suitable for the majority of ethnic groups, especially for the elder, frail patients with chronic diseases, dialysis patients with artificial blood vessels, and so on.

The remainder of this paper is arranged as follows. Section 2 presents the UCI dataset, the wearable device, participants’ demographic characteristics, the data recording conditions, and the hardware structure. The motion recognition algorithm, motion signal collection, signal normalization, data measurement method, data input format, and model framework are discussed in Section 3. Section 4 introduces the results and discussion. Conclusions are drawn in Section 5.

## 2. Experiment Result

### 2.1. Open Database

To validate the proposed algorithm, an open, large-scale database was required. The UCI dataset was determined to be suitable. The data were collected from 30 healthy participants aged 19–48 years who wore an Android smartphone (the Samsung Galaxy S2) on their waist while conducting six activities of daily living (walking, walking upstairs, walking downstairs, sitting, standing, and laying). Sensor signals measured by an accelerometer and a gyroscope were analyzed. A constant frequency of 50 Hz was used to collect data on three-axis acceleration and three-axis angular velocity. Activities were recorded by video cameras and labeled manually. The data were managed by the UCI Machine Learning Repository to ensure data quality. The accelerometer and gyroscope data were preprocessed using a noise filter. Data were sampled at 2.56 s and at a fixed sliding window with 50% overlap (128 readings/window). The accelerometer and gyroscope output three-axis values every 0.02 s. The number of data entries was 10,299, and the training and test sets were separate from the dataset itself [15]. Overall, 70% (7352 entries) and 30% (2947 entries) were used for data training and testing, respectively. From the training set, 20% (1471 entries) were used in the verification set. The recorded number of activities of daily living entries is displayed in Table 1.

### 2.2. Self-Recorded Data

#### 2.2.1. Demographics and Data Recording Criteria

**Figure 1:**
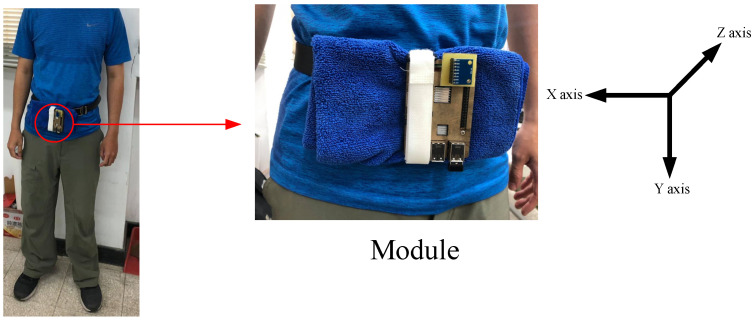
The wearable device, fitted on the waist of a participant.

The participants comprised 21 healthy male volunteers. The means ± standard deviations of their age, height, and body weight were 22 ± 2 years, 165 ± 15 cm, and 65 ± 15 kg, respectively. The number of data entries corresponding to each activity of daily living is presented in Table 2. The participants were asked to wear the wearable device on their waists (Figure 1) while performing six activities of daily living (i.e., walking, walking upstairs, walking downstairs, sitting, standing, and laying). The wearable device was composed of a Raspberry Pi 3 single-board computer (SBC), an accelerometer, and a gyroscope. The values obtained from the accelerometer and the gyroscope were converted using 2048 LSB/g and 16.4 LSB/(°/s), respectively. The direction and position of the inertial sensors were strictly controlled, with sensors along the x-, y-, and z-axes being located on the absolute right side, directly below, and the absolute front side of the body, respectively. These fixed directions and positions were used for data calibration. The researchers ensured that the sensor locations and directions were fixed to prevent the sensors from moving in the course of an activity and reducing the accuracy of the data. Specifically, the wearable device was wrapped in cloth to prevent it from becoming imbalanced. Additionally, a belt was used to fasten the wearable device on the participants’ waists to prevent it from shaking with their movements. This increased stability facilitated data collection by the accelerometer and gyroscope. The Raspberry Pi 3 SBC obtained the sensor data and saved them as txt files, which were uploaded to the cloud over Wi-Fi. Subsequently, the data were downloaded to a personal computer. A constant sampling rate of 50 Hz was adopted in collecting the three-axis acceleration and three-axis angular velocity data. A total of 13,860 data entries, each of which had 900 features, was collected. Overall, 70%, 30%, and 30% (9702, 4158, and 1941 entries) of the data were divided into the training set, test set, and verification sets, respectively. The number of data entries corresponding to each activity of daily living in the training, testing, and verification sets was 1293, 693, and 324, respectively.

#### 2.2.2. Hardware Framework

**Figure 2:**
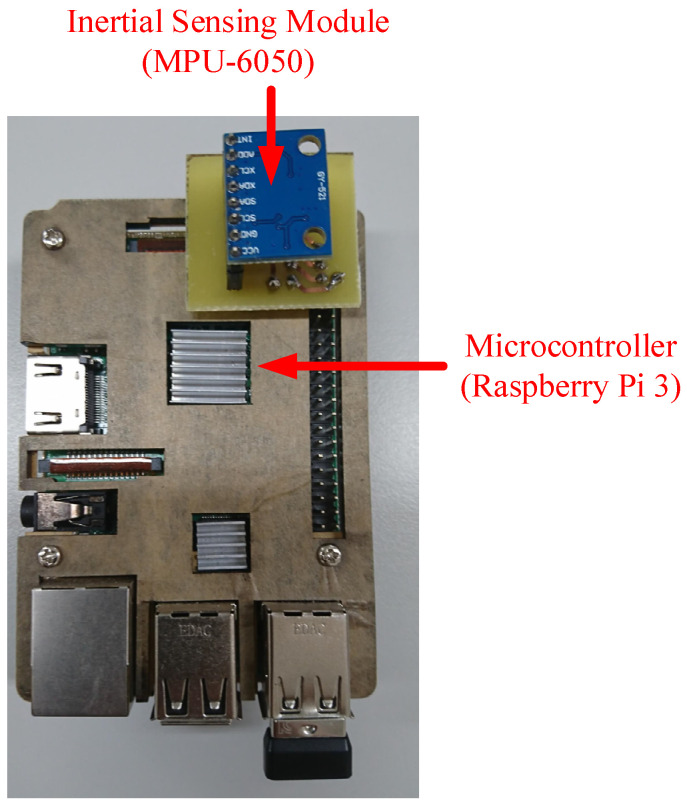
Components of the wearable device.

The wearable device consisted of a Raspberry Pi 3 SBC, six-axis inertial sensor (MPU-6050) and power bank (Figure 2). The MPU-6050 has a digital low pass filter (DLPF) for both the gyroscope and accelerometer with bandwidth of 5 Hz. It means that the low pass filter only allows lower frequencies to pass and filters out higher frequencies that come from the sensor. By using the inter-integrated circuit (I^2^C) communication protocol, the signals detected by the sensor were obtained. The acceleration range was ±2, ±4, ±8, and ±16 g. The gyroscope measured the angular velocity, which ranged from ±250 to ±500, ±1000, and ±2000 °/s. The scope and sensitivity of the accelerometer were set to ±16 g and 2048 LSB/g, respectively, and those of the gyroscope were set to ±2000 °/s and 16.4 LSB/g, respectively. A frequency of 50 Hz was employed in sampling the output signals of the accelerometer and gyroscope. The power bank provided the wearable device with a stable direct current at 5 V/2.1 A.

## 3. HAR Algorithm

Activity recognition involved activity signal collection, signal normalization, and deep-learning algorithm execution. The processes through which the algorithm was run are as follows.

### 3.1. Activity Signal Collection

**Figure 3:**
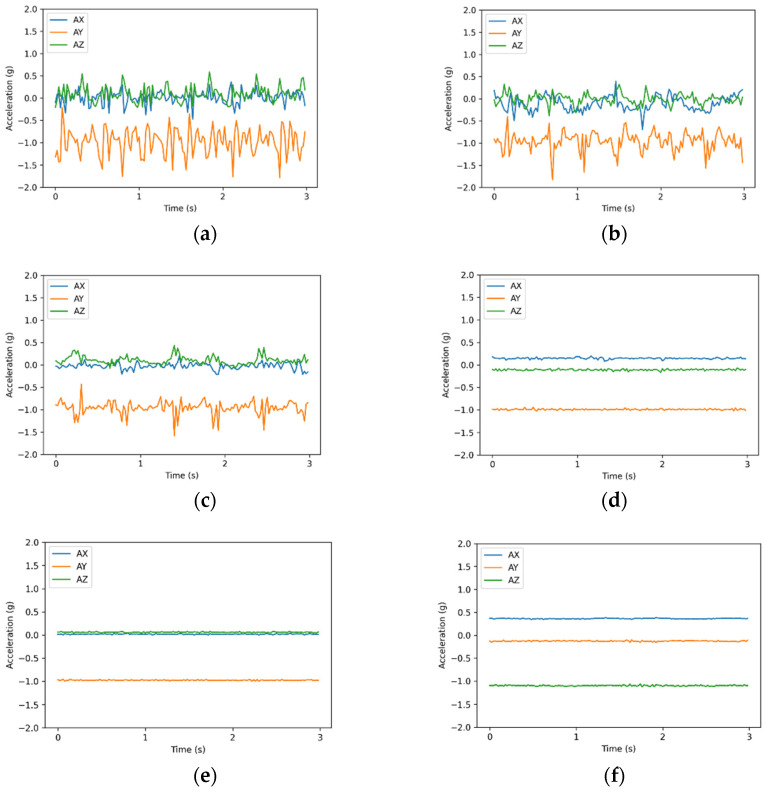
Readings from the accelerometer for each activity: (**a**) walking, (**b**) walking upstairs, (**c**) walking downstairs, (**d**) sitting, (**e**) standing, and (**f**) laying.

Figure 3 presents the accelerometer readings from each activity. AX, AY, and AZ represent the x-, y-, and z- axes of the accelerometer, respectively. Walking, walking upstairs, and walking downstairs are considered dynamic movements, whereas sitting, standing, and laying are regarded as static movements. The trends and values of activities of the same type were similar.

### 3.2. Signal Normalization

Because the precision of the accelerometer differed from that of the gyroscope, the precisions had to be calibrated to the seventh decimal place and be presented using scientific notation such that the length of each acceleration and angular velocity entry was the same. To ensure that no errors occurred when the preprocessed data were entered into the algorithm, min–max normalization was applied to limit the data to between −1 and 1.

### 3.3. Data Measurement Method and Format

The sampling rate of the wearable device was 50 Hz. Thus, a 1 × 900 matrix was obtained. The accelerator data corresponding to the x-, y-, and x-axes were designated by AX, AY, and AZ. The gyroscope data corresponding to these axes were designated as GX, GY, and GZ. Training and testing data were arranged in the same format before being entered into the classifier.

### 3.4. Network Framework

**Figure 4:**
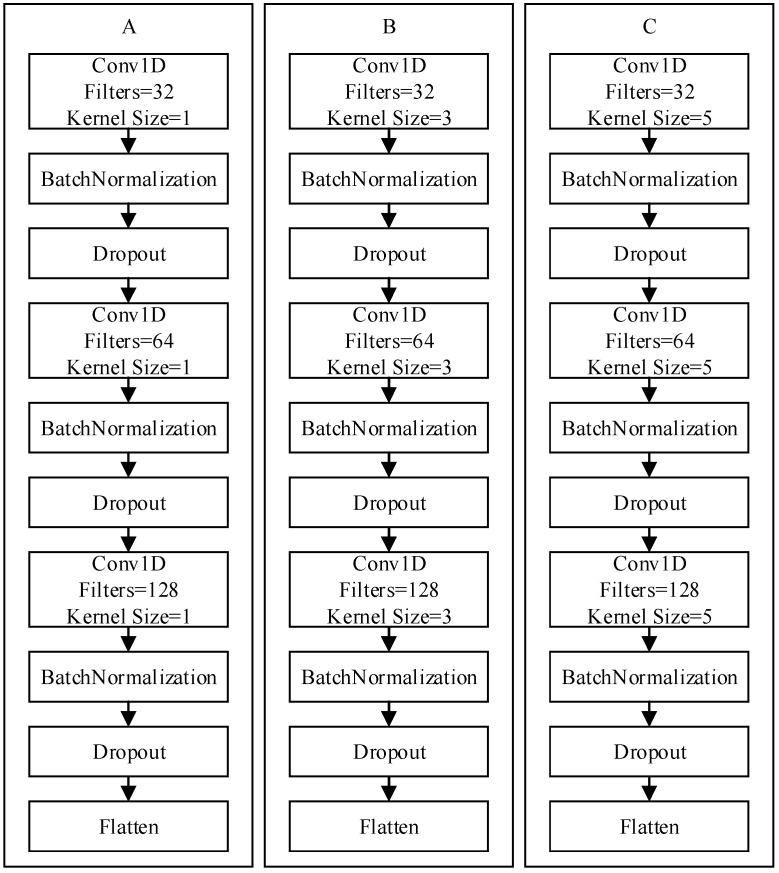
Zones A, B, and C.

**Figure 5:**
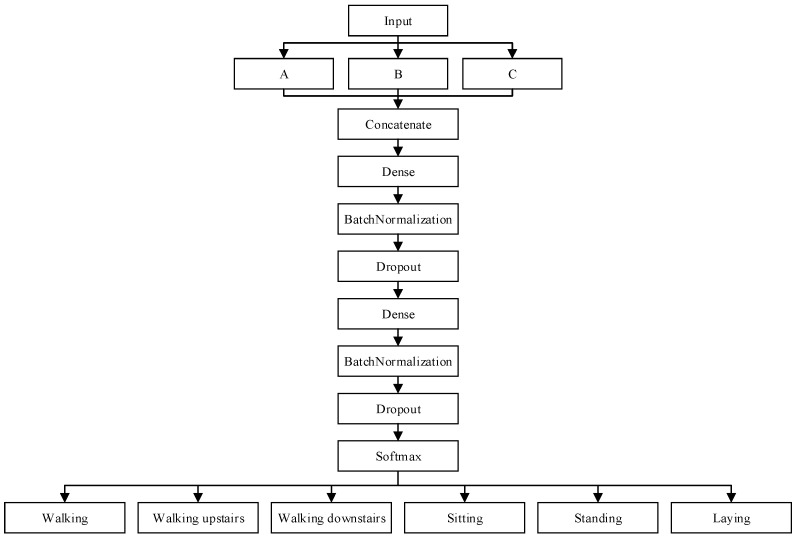
Flowchart of model procedures.

The CNN is a type of artificial neural network based on deep-learning theories. CNNs can be used in feature extraction, and through subsequent fully connected layers, they can conduct classification. This study used a feature fusion model involving kernels of varying size. After the inertial data collected by the wearable device underwent signal normalization and conversion into a fixed format, they were entered into Zone A, Zone B, and Zone C, which comprised three convolutional layers and one flattened layer (Figure 4). The three zones were concatenated and then input into a network composed of two fully connected layers. Between all layers, batch normalization was conducted, and the dropout technique was employed to prevent overfitting. The activation functions all used rectified linear units to strengthen the nonlinear relationship between network layers. It also made part of the neurons’ output equal 0, mitigating overfitting to some extent and making gradient divergence less likely to occur. Finally, the normalized exponential function SoftMax was used to calculate the probabilities of the six activities of daily living. Each action was classified into the category corresponding to its highest probability. Figure 5 presents the model framework, whereas Figure 4 displays Zones A–C.

1. Input: the six-axis data collected by the accelerometer and gyroscope.
2. Convolutional layers: In the three convolutional layers, the filters were set as 32, 64, and 128, respectively. The size of the stride was set as 1. The kernel size in Zones A, B, and C were set as 1, 3, and 5, respectively.
3. Fully connected layers: the number of neurons in the two fully connected layers was set as 256 and 512, respectively.
4. Dropout: set as 0.3 in the experiment.
5. Output: set as 6 in the experiment.
6. Optimizer and learning rate: The Adam optimizer was adopted, and the learning rate was set at 0.001. The learning rate decay was considered (minimum: 0.0000001).
7. Loss function: Categorical cross-entropy was adopted. The closer the predicted value is to the actual value, the smaller the loss function is. Conversely, the farther the predicted value from the actual value, the greater the loss function.
8. Number of iterations: In the experiment, the number of iterations was set as 1000. Early stopping was used to force the network to terminate training earlier and to save the optimal model.

## 4. Result and Discussion

The recognition performance of the proposed algorithm was examined using the assessment standard of the classification model. To demonstrate the performance of the algorithm, the following assessment indexes were used: accuracy, macro average precision, macro average recall, and *F*1 score. Their definitions are as follows:

(1) $$Accuracy(\%)=\frac{TP+TN}{TP+FP+FN+TN}\times 100\%$$

(2) $$Precision_{i}(\%)=\frac{TP_{i}}{TP_{i}+FP_{i}}\times 100\%$$

(3) $$P_{macro}(\%)=\frac{1}{n}\sum_{i=1}^{n}{Precision}_{i}$$

(4) $$Recall_{i}(\%)=\frac{TP_{i}}{TP_{i}+FN_{i}}\times 100\%$$

(5) $$R_{macro}(\%)=\frac{1}{n}\sum_{i=1}^{n}{Recall}_{i}$$

(6) $$F1-Score(\%)=2\times \frac{P_{macro}\times R_{macro}}{P_{macro}+R_{macro}}$$

where *TP* is a true positive, *TN* is a true negative, *FP* is a false positive and *FN* is a false negative. *i* denotes the activity categories (*i* = 1–6).

With *k* set at 10 such that the data were divided into 10 equal parts, *k*-fold cross-validation was conducted. For training, *k*−1 was used, with the 1 deducted used for testing. The researchers ensured that testing data were excluded from the training set; they were to be used for model assessment.

### 4.1. Assessment Indexes of the UCI Dataset

In this study, the accuracy of the algorithm on the UCI dataset was 97.49%. *P_macro_* was 97.64%, *R_macro_* was 97.46%, *F*1-*score* was 97.51%, and the tenfold cross-validation accuracy was 99.56%. Additionally, the comparisons of the proposed method are listed in the Section 4.7.

### 4.2. Confusion Matrix of the UCI Dataset

**Figure 6:**
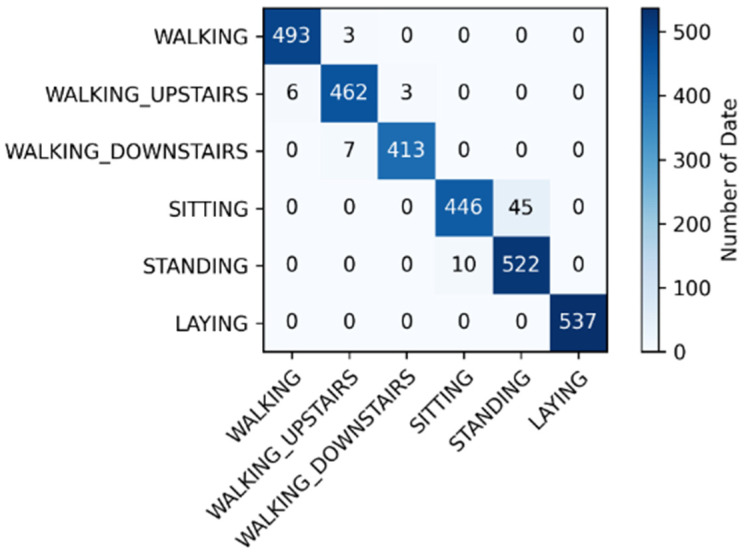
Confusion matrix of the open dataset.

Figure 6 indicates that the dynamic movements (walking, walking upstairs, and walking downstairs) were easily misclassified, whereas the static movements (sitting, standing, and laying) were not. This is because when the participants lay down, the gyroscope values changed substantially. As for sitting and standing, classification errors occurred easily because the data characteristics of the two were similar. Moreover, the sensors did not exhibit obvious changes when the participants were in these two positions.

### 4.3. Model Accuracy and Loss Function of the UCI Dataset

**Figure 7:**
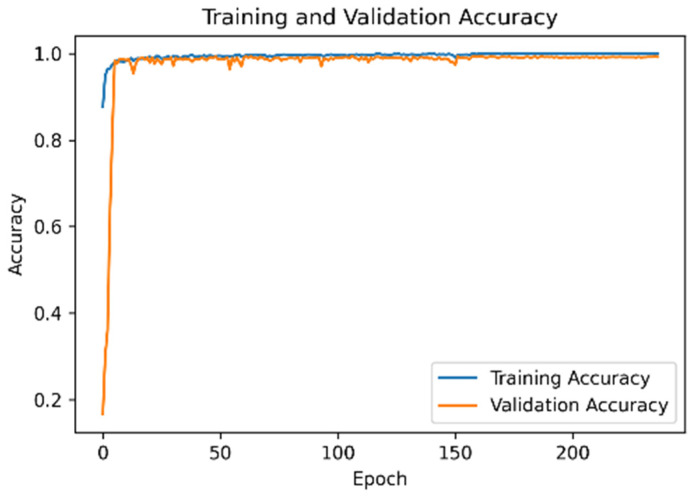
Model accuracy of the UCI dataset.

**Figure 8:**
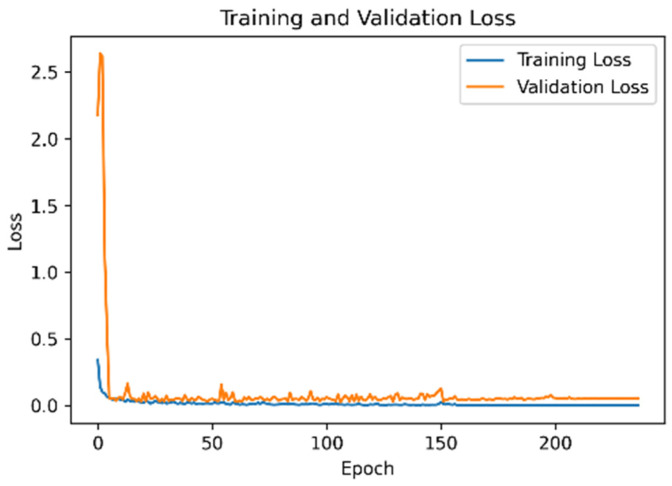
Model loss function of the UCI dataset.

Figure 7 is Model accuracy and Figure 8 is mode loss demonstrate that when the proposed network framework was applied to the UCI dataset, some fluctuations were observed in the early stage of model training. However, the accuracies of the training and verification sets reached 97%. After the learning rate decay technique was employed, the accuracy and the loss function converged.

### 4.4. Various Assessment Indexes of Self-Recorded Data

The accuracy of the algorithm in classifying the activities from the self-recorded data was 96.27%. The *P_macro_* was 96.39%, the *R_macro_* was 96.27%, the *F*1-*score* was 96.26%, and the tenfold cross-validation accuracy was 97.46%.

### 4.5. Confusion Matrix of the Self-Recorded Data

**Figure 9:**
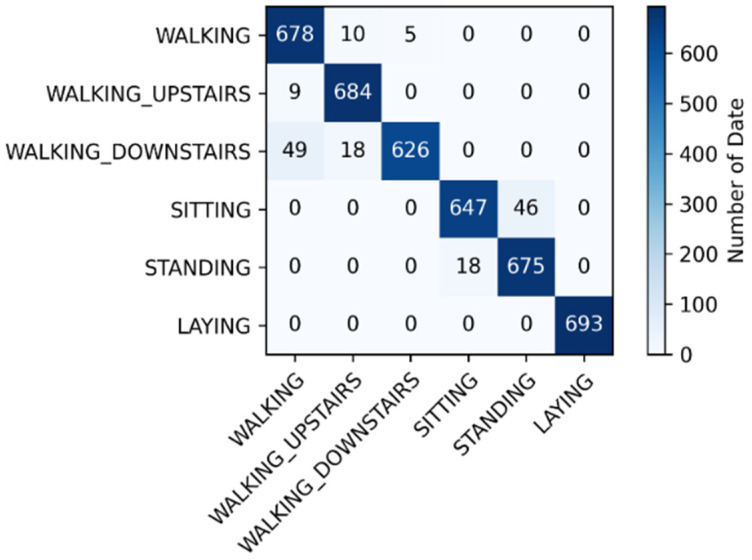
Confusion matrix of the self-recorded data.

As shown in Figure 9, the classifications for the activity of laying were all accurate. This is because the gyroscope detected change substantially when the participants were in this position. However, the remainder of the data did not exhibit as high an accuracy for dynamic movements as they did in the UCI dataset. This may be because the self-recorded data did not undergo filtering or denoising by algorithms; only kernels of varying size were used for feature extraction. Consequently, the accuracy corresponding to the self-recorded data was lower than that of the UCI dataset, but only slightly (96.27% vs. 97.49%).

### 4.6. Model Accuracy and Loss Function of the UCI Dataset

**Figure 10:**
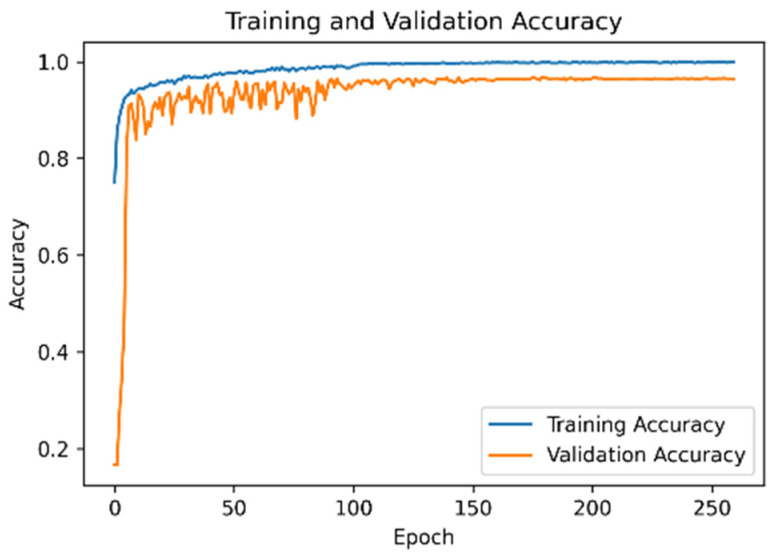
Model accuracy of the self-recorded data.

**Figure 11:**
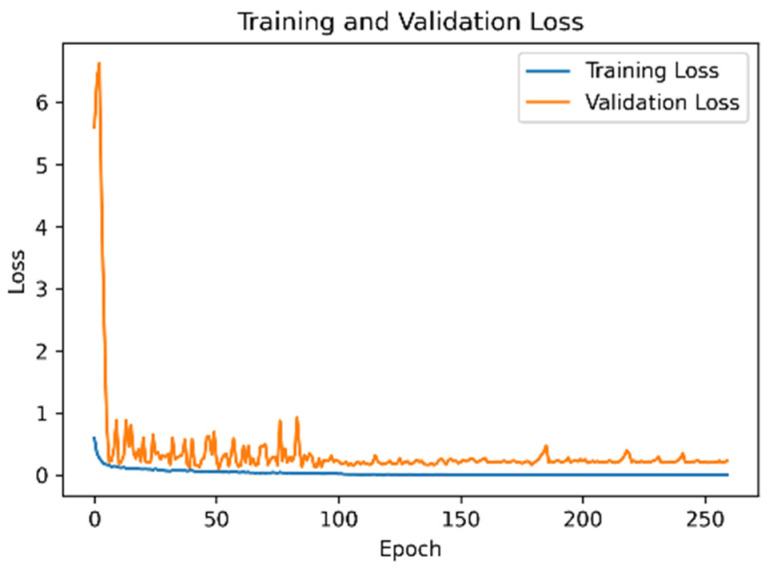
Model loss function of the self-recorded data.

Figure 10 and Figure 11 reveal that after 100 training iterations, the accuracies of the verification set and the model were both stabilized.

### 4.7. Accuracy of the UCI Dataset

Table 3 presents a comparison of classification accuracy obtained using the UCI dataset for training and testing in relevant studies. The present experimental results were generally superior to those reported in [6,16,18,19,20,21,23,24,25] but were comparable to those in [24]. Regarding model frameworks, because neither GRUs nor the LSTM were employed, the complexity of the present algorithm is slightly lower than that of the algorithm in [24]. The data in [22] were richer. In that study, features were both manually extracted and extracted using a CNN. Subsequently, feature fusion was conducted, yielding results that were more favorable than the present results. By contrast, in this study, features were extracted only using kernels of varying size, but the accuracy achieved was also favorable (97.49%). A classification accuracy of 100% was achieved in [26]. However, moderate and coarse network parameters could not be employed to construct an identical network framework. Despite these differences, ref [26] was included in the present comparison because that study also used the UCI dataset. Herein, the application of tenfold cross-validation to the UCI dataset achieved an extremely high accuracy of 99.56%. This is because the data division method of the database source was not used; rather, data were merged and subjected to a tenfold cross-validation. If the database sources were not used for data division, an accuracy comparable to that obtained through cross-validation could have been achieved.

## 5. Conclusions

In general, the sensing device is designed to wear on the hands. When the hands of patients with artificial blood vessels cannot move freely, the accuracy of activity recognition will be lower than that of normal people. Therefore, the proposed method, which collects data from the six-axis inertial sensor at the waist, is more flexible, and it can accurately recognize the six daily activities for specific patients. HAR is a complex topic. In this study, a model with three parallel CNN networks was proposed. The networks were concatenated to achieve a feature fusion model with kernels of varying size. This model was applied in HAR with regard to data from the public UCI dataset and from self-recorded data on six activities of daily living performed by 21 participants. The classification accuracies of the UCI dataset and the self-recorded data were 97.49% and 96.27%, respectively. In tenfold cross-validation, the classification accuracies were 99.56% and 97.46%, respectively. Furthermore, the sensors can be installed on more parts of the subject, such as head, hands, and feet, to collect more data and use data fusion technology with convolutional neural network to recognize more movements.

## Figures and Tables

**Table 1:** Number of entries in each dataset in the UCI dataset.

	Training Set	Test Set	Verification Set	Total
Walking	981	496	245	1722
Walking upstairs	858	471	215	1544
Walking downstairs	789	420	197	1406
Sitting	1029	491	257	1777
Standing	1099	532	275	1906
Laying	1125	537	282	1944

**Table 2:** Participant characteristics.

Parameters	Data Set
Men	21
Age (years)	22 ± 2
Height (cm)	165 ± 15
Weight (kg)	65 ± 15

**Table 3:** Comparison of accuracy obtained using the UCI dataset in various studies.

Examined Studies	Accuracy
Yen et al. [6]	95.99%
Xia et al. [16]	95.78%
Yang et al. [18]	95.89%
Mutegeki and Han [19]	92.13%
Nidhi et al. [20]	96.20%
Hernández et al. [21]	92.67%
Thakur and Biswas [22]	99.10%
Ullah et al. [23]	93.13%
Nafea et al. [24]	97.05%
Xu et al. [25]	94.50%
Avilés-Cruz et al. [26]	100%
Ours	97.49%

## Data Availability

We exclude this statement because of the study did not report any data.
